# Pressure and Temperature Dependence of Optical Resonance Wavelength (ORW) of Large-Element Surface-Micromachined Optical Ultrasound Transducers (SMOUTs)

**DOI:** 10.3390/s26020480

**Published:** 2026-01-11

**Authors:** Kaustubh Upadhyay, Cheng Fang, Zhiyu Yan, Xuan Li, Jun Zou

**Affiliations:** Department of Electrical and Computer Engineering, Texas A&M University, College Station, TX 77843, USA; kaustamu@tamu.edu (K.U.); fangchengok2007@tamu.edu (C.F.); yan1383@tamu.edu (Z.Y.); lixuan202366@tamu.edu (X.L.)

**Keywords:** optical resonance wavelength (ORW), surface-micromachined optical ultrasound transducer (SMOUT), ambient pressure, temperature

## Abstract

This paper investigates optical resonance wavelength (ORW) shifts in large-element, fiber-tip surface-micromachined optical ultrasound transducers (SMOUTs) induced by changes in ambient pressure and temperature. The displacement behavior of the SMOUT top membrane under varying pressure and temperature conditions is analyzed and modeled, and simulation results are presented for fiber-tip SMOUTs with four diameters (200, 400, 600, and 800 µm). Fabricated and assembled fiber-tip SMOUTs are experimentally characterized using two dedicated setups to measure their reflectivity spectra and ORW shifts over ambient pressures from 80 kPa to 120 kPa and temperatures from 25 °C to 45 °C. The experimental data show good agreement with the simulation results. These findings provide a solid basis for active control and compensation of ORW shifts via pressure and temperature adjustment. By stabilizing the reflectivity spectrum and minimizing ORW drift, the use of non-tunable high-power light sources to interrogate arrays of fiber-tip SMOUTs with enhanced operational stability and sensitivity is enabled.

## 1. Introduction

Recently, optical ultrasound transducers (OUTs) with high acoustic sensitivity and small form factors have been demonstrated, including micro-ring resonators (MRRs) [1,2], Bragg grating waveguides (BGWs) [3,4], and Fabry–Perot interferometers [5]. However, MRR- and BGW-based OUTs typically require side coupling, which would be difficult to implement and could impede the detection of incoming ultrasound waves. In contrast, FPI-based OUTs can be directly interfaced with optical fibers and are therefore suitable for a wide range of applications. Most reported FPI-based OUTs [6,7,8,9,10], however, are individually assembled with relatively complex structures, which limits scalability for mass production and reduces device-to-device uniformity for array implementations.

More recently, surface-micromachined optical ultrasound transducers (SMOUTs) have been developed with good sensitivity (down to Pascal level), high element density (element diameters from tens to hundreds of micrometers), and wideband acoustic reception [11,12,13,14]. Because they are micromachined, these devices offer improved uniformity and are more amenable to batch fabrication of large arrays for photoacoustic computed tomography (PACT). In addition, the SMOUT diameter and its frequency response can be easily tailored to match the acoustic center frequency (*f*_c_) of the generated PA signals.

**Figure 1:**
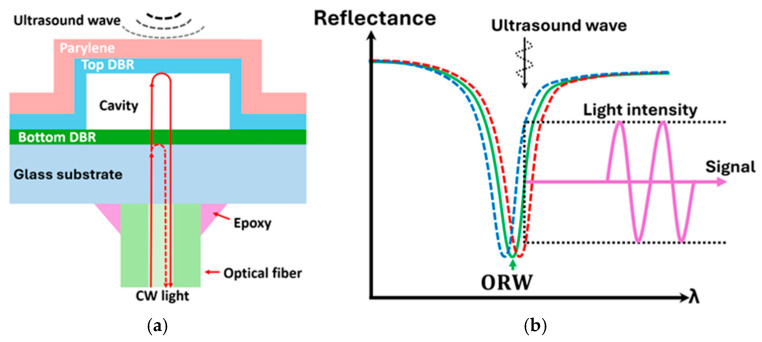
Diagrams of (**a**) the cross-section design of the fiber-tip SMOUT, and (**b**) the SMOUT reflectivity spectrum and ORW shifted by the top diaphragm vibration upon ultrasound wave and the modulated light intensity as the output signal, where the original, positively- and negatively-shifted spectra are indicated by the solid-green, dashed-red, and dashed-blue curves, respectively.

To further optimize the reliability, flexibility, and ease of integration for PACT applications, the SMOUT is usually interfaced with a multimode optical fiber. As shown in Figure 1a, the SMOUT—realized as a hollow F-P cavity formed by two distributed Bragg reflectors (DBRs)—is assembled on the fiber tip and fixed with epoxy. A Parylene layer is coated on the top DBR to attenuate high-order vibration modes and protect the SMOUT from scratches, mechanical damage, and contamination. When continuous-wave (CW) light is delivered to the cavity through the optical fiber, the wavelength corresponding to a local minimum reflectivity is defined as the optical resonance wavelength (ORW). When ultrasound waves impinge on the SMOUT, the top diaphragm (top DBR + Parylene) vibrates, shifting the ORW and modulating the intensity of the light reflected back into the optical fiber, which is then used as the output signal (Figure 1b).

**Figure 2:**
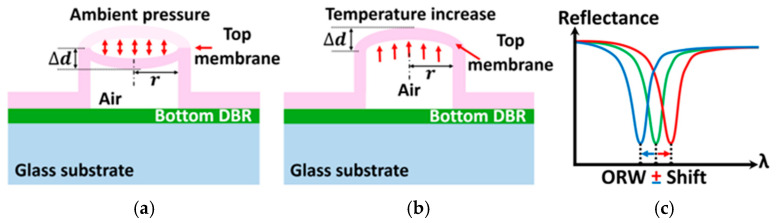
Diagrams of the top membrane deflections due to (**a**) ambient pressure variation and (**b**) temperature increase. (**c**) ORW shifts due to pressure and temperature change, where the original, positively- and negatively-shifted spectra are indicated by the solid-green, red, and blue curves, respectively.

To enable the SMOUT to operate in liquids (such as water) for acoustic reception, its cavity must be sealed. Small-element SMOUTs (ϕ ≤ 100 µm) can be vacuum-sealed, e.g., using low-temperature oxide (LTO) deposited by low-pressure chemical vapor deposition (LPCVD) [12,14], to provide proper resistance to ORW shifts due to changes in ambient pressure or temperature. However, this vacuum-sealing approach is not suitable for large-element SMOUTs (ϕ ≥ 200 µm), because the large pressure difference between the inside and outside of the cavity causes the top membrane to collapse onto the bottom DBR. Consequently, large-element SMOUT cavities are instead filled with air/gas and sealed with waterproof glue. Variations in ambient pressure and temperature (Figure 2a,b) can then compress or expand the trapped gas, changing the cavity length (i.e., the separation between the top and bottom DBRs) and shifting the ORW (Figure 2c). This can severely degrade the ultrasound detection performance of the SMOUT, particularly when the interrogation wavelength drifts outside the operational range. Therefore, accurate analysis and quantification of ORW shifts of large-element SMOUTs under different ambient pressure and temperature conditions are essential for the control and stabilization of the ORW.

This paper reports a systematic study of the pressure and temperature dependence of the ORW shifts in large-element fiber-tip SMOUTs. First, the underlying principles governing the dependence of the top membrane deflection on ambient pressure and temperature are described. SMOUTs with diameters of 200, 400, 600, and 800 µm are modeled and simulated using COMSOL Multiphysics (v. 6.3) and MATLAB (v. R2024b) to investigate the membrane displacement under pressure and temperature changes and the resulting reflectivity spectrum shifts. Next, the fabrication of a SMOUT element and its assembly onto the tip of a multimode optical fiber are presented. Two experimental setups are then employed to characterize the spectral shifts of the four-diameter fiber-tip SMOUTs over pressure and temperature ranges of 80–120 kPa and 25–45 °C, respectively. Finally, conclusions and discussions are provided. These results establish a foundation for actively controlling pressure and temperature to compensate ORW shifts; with a stabilized spectrum and ORW, it becomes feasible to use non-tunable high-power light sources to achieve optimal SMOUT stability and sensitivity.

## 2. Principle and Simulation

**Figure 3:**
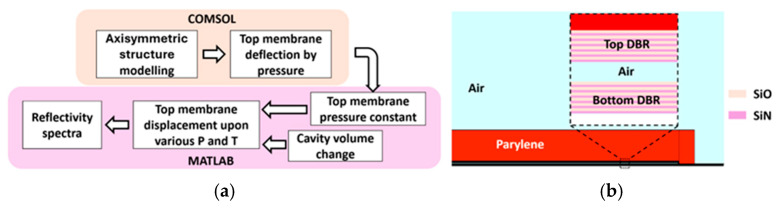
Diagrams of (**a**) the simulation process flow and (**b**) the 2D FEM model of the SMOUT in COMSOL.

Figure 3a shows the overall flow of the simulation process. When an ambient pressure $P_{a}\;$is applied to the SMOUT (Figure 2a), the deflection ∆d at the center of the top membrane can be expressed as [15]:

(1) $$∆d=\frac{P_{a}r^{4}}{64D}$$

where r is the radius of the top membrane, and D is the equivalent flexural rigidity, determined by the Young’s modulus, thickness, and Poisson’s ratio of the multilayer top membrane. Accordingly, the pressure–deflection relationship can be written as:

(2) $$P_{a}=\frac{64D}{r^{4}}∆d=k∆d$$

where k is defined as the equivalent pressure constant of the top membrane. To investigate k, a 2-D axisymmetric finite element (FEM) model is built (Figure 3b) in COMSOL Multiphysics (Solid Mechanics module) to simulate the deformation of the top membrane. By applying different ambient pressures to the SMOUT top membrane, the slope of the simulated pressure–deflection curve at the membrane center is extracted as k. The material properties and geometric parameters used in the simulations are summarized in Table 1. The relatively large variation in parylene thickness (13.4–19 µm) primarily arises because the SMOUTs with different diameters were not coated with parylene in the same deposition batch, resulting in non-uniform thickness. However, for each SMOUT, the parylene thickness used in the simulation matches the experimentally measured thickness of the actual device.

For a sealed SMOUT with an initial pressure $(P_{0}$) inside the cavity and an additional ambient pressure $P_{a}$ applied onto its top membrane, the pressure inside the cavity at equilibrium will be slightly lower than $P_{0}+P_{a}$ due to the restoring effect of the deformed membrane, which is represented by an equivalent restoring pressure (P=k∆d). Thus,

(3) $$\left(P_{0}+P_{a}-k∆d\right)\left(V_{0}-∆V\right)=nRT=P_{0}V_{0}$$

where $V_{0}$ is the initial (undeformed) volume of the SMOUT cavity and ∆V is the cavity volume change due to membrane deformation, obtained by integrating the out-of-plane displacement over the entire top membrane. After calculating ∆V and solving (3) in MATLAB, the central deflection (∆d) of the top membrane is obtained. The new cavity length is then calculated by subtracting the ∆d from the original gap size.

**Figure 4:**
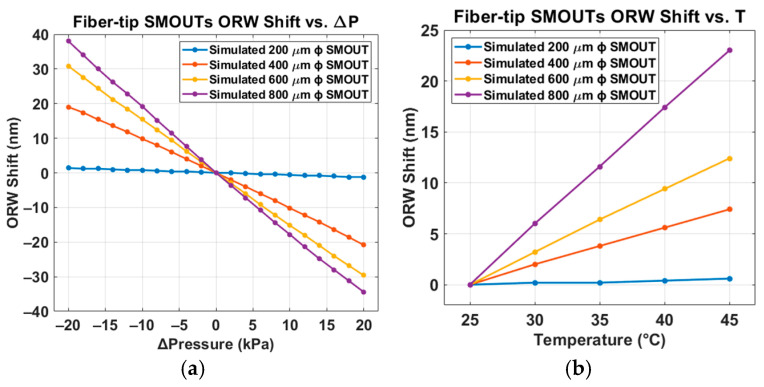
Simulated ORW shifts of four large-element fiber-tip SMOUTs with different diameters under (**a**) ambient pressure variation from 80 kPa to 120 kPa and (**b**) temperature variation from 25 °C to 45 °C.

With the thicknesses and refractive indices of the individual layers forming the DBRs and substrate, the reflectivity spectrum of the SMOUT is computed in MATLAB via the transfer-matrix method (TMM) [17]. The optical stack includes the glass substrate, the thin-film layers in the bottom DBR, the air gap (cavity), and the thin-film layers in the top DBR. Upon the temperature increase (Figure 2b), the pressure inside the SMOUT cavity increases proportionally according to the ideal gas law, and the reflectivity spectrum can be obtained using a similar procedure as for the ambient-pressure case based on Equation (4). The simulated ORW shifts for four-diameter SMOUTs under varying ambient pressure (80–120 kPa) and temperature (25–45 °C) are summarized in Figure 4a and Figure 4b, respectively.

(4) $$\left(P_{0}-k∆d\right)\left(V_{0}+∆V\right)=nR(T+∆T)$$

## 3. Experimental Setup

### 3.1. Fiber-Tip SMOUT Fabrication and Assembly

The fabrication procedure of the SMOUT is similar to that reported previously [12,14]. First, the bottom DBR (several pairs of SiN/SiO) is deposited on a glass substrate, followed by the deposition and patterning of a sacrificial layer. Second, several pairs of SiN/SiO are deposited on the patterned sacrificial layer to form the top DBR and create a SMOUT array. Third, the array is diced into individual elements, which are then immersed in an acid solution to wet-etch the sacrificial layer and release the top DBR. Finally, a Parylene layer is coated on the top DBR.

**Figure 5:**
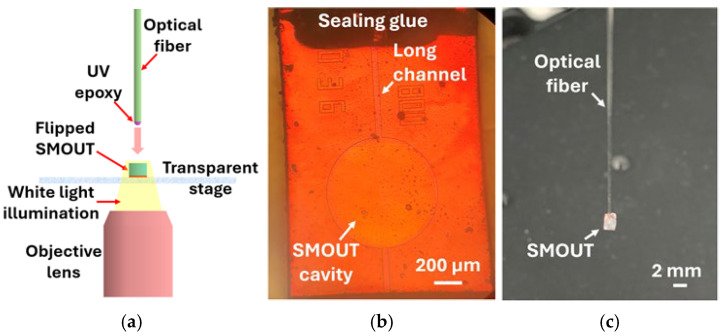
(**a**) Schematic of the assembly of the SMOUT onto an optical fiber with the aid of an inverted microscope. Photographs of a representative fiber-tip SMOUT with 800 µm diameter in (**b**) front view and (**c**) oblique view.

After fabrication, each SMOUT is assembled onto the tip of a 200 µm diameter optical fiber (Thorlabs, Newton, NJ, USA) with the aid of an inverted microscope (Nikon, Tokyo, Japan) (Figure 5a). The fiber core is aligned with the SMOUT center to optimize light coupling efficiency and thereby maximize the acoustic sensitivity. The downward-oriented optical fiber, with ultraviolet (UV) epoxy (Norland, Jamesburg, NJ, USA) applied to its tip, is lowered onto the glass substrate of the flipped SMOUT, followed by UV curing. Since the vent hole at the end of the long channel is opened after Parylene coating, it is subsequently sealed with waterproof glue (Figure 5b) to complete the SMOUT-fiber assembly. Due to the tensile stress, the overall deflection of the top membrane (with a diameter of 200–800 µm) is much less than 1 µm. As a result, hysteresis and aging/creep are expected to be very small or negligible. The silicone glue used to seal the cavity showed little shrinkage during the curing, resulting in minimal effects on the initial cavity pressure after the sealing. The length, width, and height of each SMOUT are 2.26 mm, 1.6 mm, and 0.7 mm, respectively. The front and oblique views of a representative fiber-tip SMOUT with 800 µm diameter are shown in Figure 5b and Figure 5c, respectively.

### 3.2. Ambient Pressure Testing

**Figure 6:**
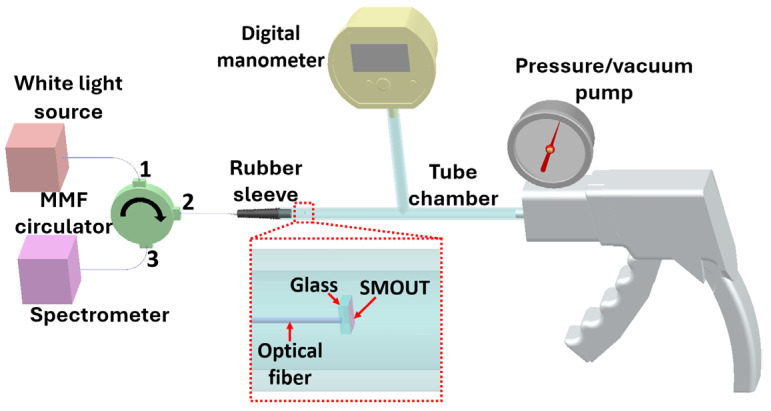
Schematic of the experimental setup used to investigate SMOUT ORW shifts under varying ambient pressure. MMF: multimode fiber.

An experimental setup (Figure 6) is built to investigate the reflectivity spectrum and ORW shifts of the fiber-tip SMOUT under varying ambient pressure. White light (OceanOptics, Orlando, FL, USA) is delivered to the SMOUT through a multimode fiber (MMF) circulator (Thorlabs, Newton, NJ, USA) from Port 1 to Port 2, and the light reflected by the SMOUT propagates from Port 2 to Port 3 and is detected by a spectrometer (OceanOptics, Orlando, FL, USA). The fiber-tip SMOUT is encapsulated in a sealed rubber sleeve and inserted into one port of a T-shaped tube chamber, with the other two ports connected to a hand-held pressure/vacuum pump (Mityvac, St. Louis, MO, USA) and a digital manometer. The chamber pressure is adjusted from 80 kPa to 120 kPa (i.e., ±20 kPa around 1 atm) in 2 kPa steps. At each pressure step, three spectra are recorded, each obtained with 64-time averaging, to determine the mean value and deviation of the ORW.

The measured spectrum $S_{SMOUT}$ is not the reflectivity spectrum of the fiber-tip SMOUT alone, because the white-light source has a nonuniform spectrum and there are additional reflections from other optical components (e.g., circulator, fibers). To extract the SMOUT reflectivity spectrum ($R_{SMOUT}$), two additional measurements are performed. First, with no device on the fiber tip (before assembly), the captured spectrum $S_{background}$ represents the background reflections from all components except the SMOUT, so the contribution from the SMOUT alone is $S_{SMOUT}-S_{background}$. Second, with a mirror (assumed ideal) placed on the fiber tip, the captured spectrum $S_{mirror}$ is the sum of $S_{background}$ and the mirror-reflected light. As a result, the light incident onto mirror is $S_{mirror}-S_{background}$, which can be treated as the light incident onto SMOUT. Therefore, $R_{SMOUT}$ can be expressed by

(5) $$R_{SMOUT}=\frac{S_{SMOUT}-S_{background}}{S_{mirror}-S_{background}}$$

### 3.3. Temperature Testing

**Figure 7:**
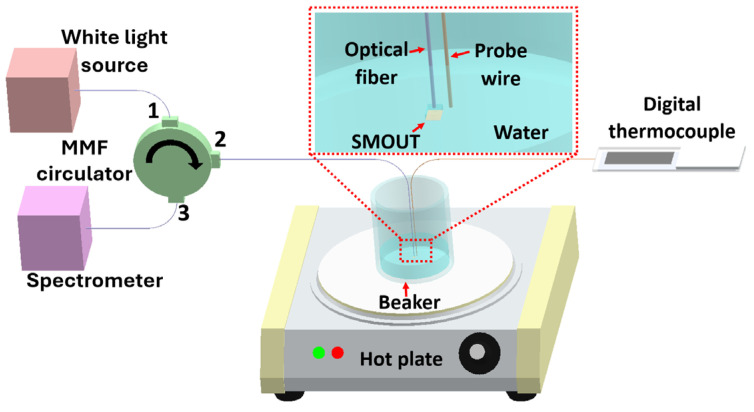
Schematic of the experimental setup used to investigate SMOUT ORW shifts under temperature variation. MMF: multimode fiber.

A similar setup is used to investigate the spectrum and ORW shifts of the fiber-tip SMOUT (without encapsulation) under temperature variation (Figure 7). A hot plate (VWR, Radnor, PA, USA) is used for heating, and a digital thermocouple with a probe wire monitors the temperature. To avoid contaminating or damaging the SMOUT from the probe wire, the fiber-tip SMOUT and the probe wire are both immersed in the water and placed in close proximity. Owing to the high thermal conductivity of the water, the temperature measured by the probe wire can be used to estimate that of the SMOUT. The temperature is increased stepwise from 25 °C to 45 °C in increments of 5 °C. At each temperature step, three spectra are recorded after a 10 min stabilization period, each with 64-time averaging, to determine the mean value and deviation of the ORW.

## 4. Experimental Results

### 4.1. ORW Shift Under Different Ambient Pressure

**Figure 8:**
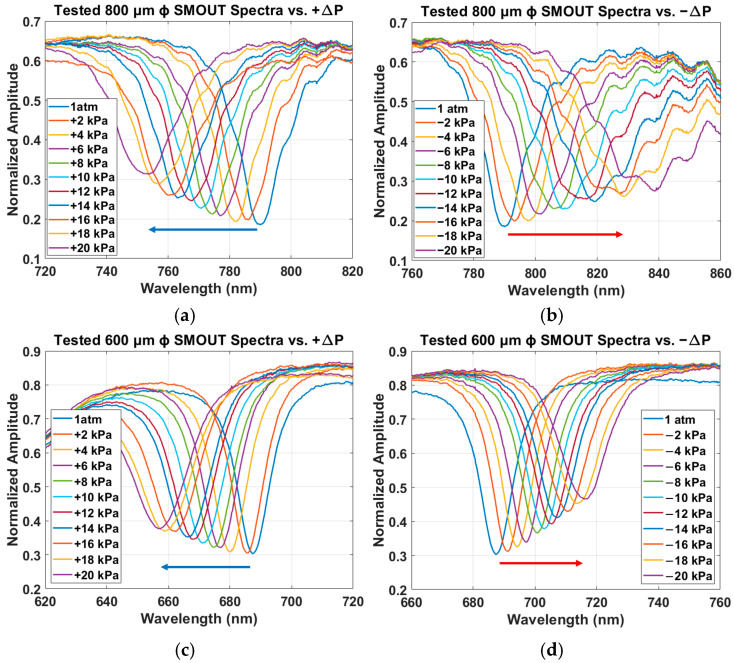

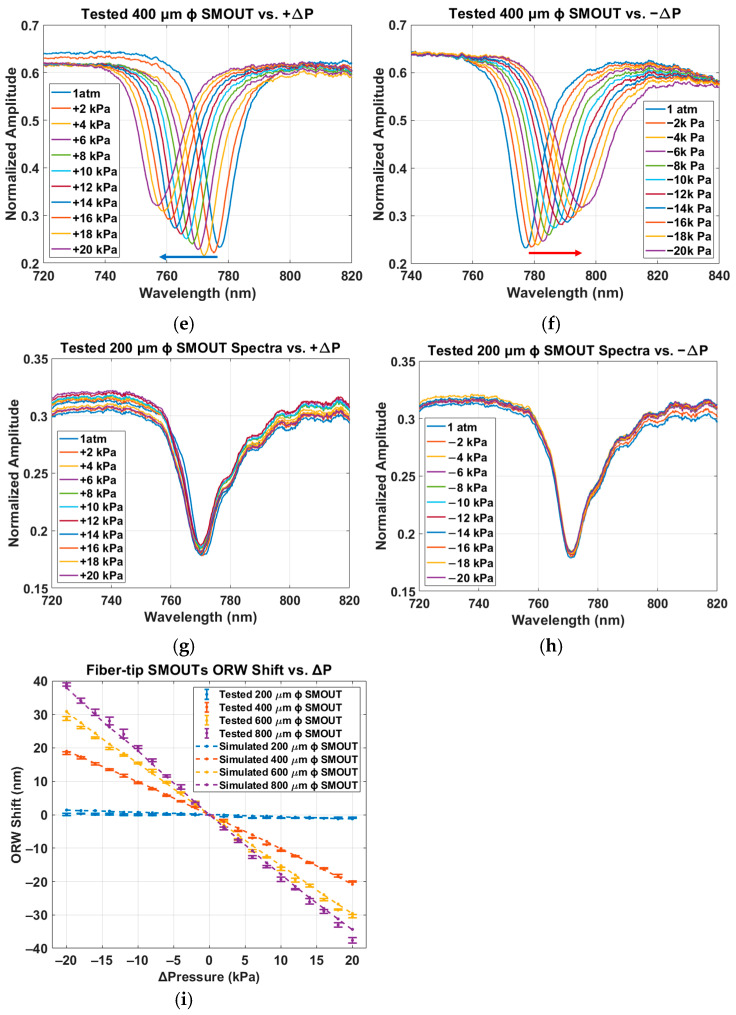
Measured reflectivity spectra of fiber-tip SMOUTs with diameters of 800 µm under (**a**) +Δ*P* and (**b**) −Δ*P*; 600 µm under (**c**) +Δ*P* and (**d**) −Δ*P*; 400 µm under (**e**) +Δ*P* and (**f**) −Δ*P*; and 200 µm under (**g**) +Δ*P* and (**h**) −Δ*P*, where the negative and positive ORW shifts are indicated by blue and red arrows, respectively. (**i**) Comparison of measured and simulated ORW shifts of the four-diameter SMOUTs as a function of ambient pressure change, with *R*^2^ values 0.9970, 0.9962, 0.9984 for the 800, 600, and 400 µm ϕ SMOUTs, respectively.

The reflectivity spectra of the four large-element SMOUTs (200, 400, 600, and 800 µm in diameter) under different ambient pressures are shown in Figure 8a–h. The wavelength range of the horizontal axis is kept identical across all plots to better compare the ORW shifts. Negative and positive ORW shifts are indicated by blue and red arrows, respectively. No arrows are shown in Figure 8g,h because the ORWs exhibit negligible shifts. For the 800 µm diameter SMOUT under negative Δ*P* (Figure 8b), the quality factor (*Q*) decreases as the magnitude of pressure and ORW shift increase, where the absolute value of the slope and the corresponding sensitivity upon Δ*P* = −20 kPa is around 78% of that upon Δ*P* = 0 kPa. This degradation is attributed to the larger deflection of the top membrane, which weakens the optical reflection and interference within the cavity. As the ambient pressure changes from 120 kPa (Δ*P* = +20 kPa) to 80 kPa (Δ*P* = −20 kPa), the ORWs of the 800, 600, 400, and 200 µm diameter SMOUTs increase approximately linearly, with sensitivities of about 1.91, 1.48, 0.96, and 0.03 nm/kPa, respectively. The measured ORW shifts versus Δ*P* for the four-diameter SMOUTs, together with the simulated results from Figure 4a, are summarized in Figure 8i (the mean values and deviations of the ORW shifts are indicated by the center dots and error bars, respectively), showing reasonably good agreement, with *R*^2^ values of 0.9970, 0.9962, and 0.9984 for the 800, 600, and 400 µm ϕ SMOUTs, respectively. The *R*^2^ value of the 200 µm ϕ SMOUT is not presented, because the ORW shifts are extremely small and the comparison is not that meaningful.

### 4.2. ORW Shift Under Different Temperature Increase

**Figure 9:**
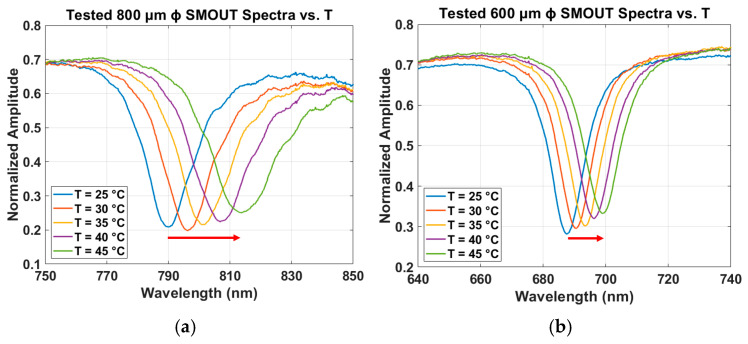

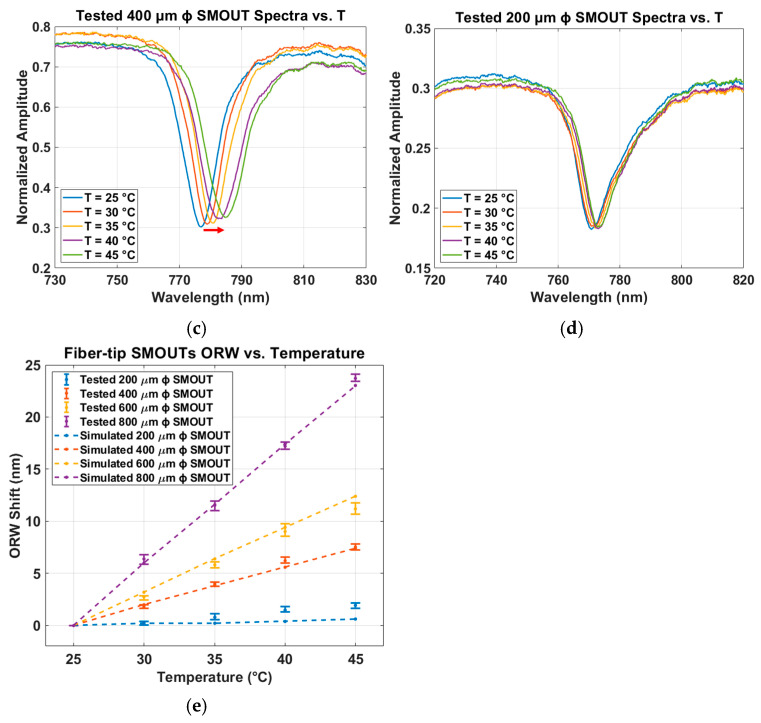
Measured reflectivity spectra of fiber-tip SMOUTs with diameters of (**a**) 800 µm, (**b**) 600 µm, (**c**) 400 µm, and (**d**) 200 µm under increasing temperature, where the positive ORW shifts are indicated by the red arrows. (**e**) Comparison of measured and simulated ORW shifts of the four SMOUT diameters as a function of temperature, with *R*^2^ values of 0.9980, 0.9739, and 0.9879 for the 800, 600, and 400 µm ϕ SMOUTs, respectively.

The measured reflectivity spectra of the four large-element SMOUTs (200, 400, 600, and 800 µm in diameter) under different temperature increases are shown in Figure 9a–d. Positive ORW shifts are indicated by red arrows; no arrow is shown in Figure 9d because the ORW change is minimal. Since the ORW shifts are relatively small, the quality factors (*Q*) of the resonances remain nearly unchanged. As the temperature increases from 25 °C to 45 °C, the ORWs of the 800, 600, 400, and 200 µm diameter SMOUTs increase approximately linearly, with sensitivities of about 1.19, 0.56, 0.37, and 0.097 nm/°C, respectively. The measured ORW shifts versus temperature for the four-diameter SMOUTs, together with the simulated results from Figure 4b, are summarized in Figure 9e (the mean values and deviations of the ORW shifts are indicated by the center dots and error bars, respectively), showing reasonably good agreement, with *R*^2^ values of 0.9980, 0.9739, and 0.9879 for 800, 600, and 400 µm ϕ SMOUTs, respectively. Again, the *R*^2^ value of the 200 µm ϕ SMOUT is not presented, because the ORW shifts are extremely small and the comparison is not that meaningful.

## 5. Conclusions and Discussion

The pressure and temperature dependence of the ORW of large-element fiber-tip SMOUTs has been analyzed and quantified. Using COMSOL and MATLAB, the reflectivity spectra and ORW shifts of the SMOUTs were modeled and simulated. In addition, two experimental setups were developed to measure the spectra and ORW shifts under varying ambient pressure and temperature, respectively. The experimental results show good agreement with the simulations over pressure and temperature variations comparable to those typically encountered in clinical settings.

These outcomes establish a solid foundation for actively controlling and compensating ORW shifts via pressure and temperature adjustment. By stabilizing the reflectivity spectrum and minimizing ORW drift, it becomes feasible to use non-tunable high-power light sources to interrogate arrays of fiber-tip SMOUTs with improved operational stability and sensitivity for a variety of acoustic tomographic imaging applications.

## Figures and Tables

**Table 1:** Material Properties and Geometric Parameters Used in the Simulation.

Material	Young’s Modulus	Poisson’s Ratio	Density	Thickness
Silicon Oxide	72.9 GPa	0.165	$2219\;\mathrm{kg}/m^{3}$	130.2 nm
Silicon Nitride	250 GPa	0.23	$3100\;\mathrm{kg}/m^{3}$	87.5 nm
Parylene	2.8 GPa [16]	0.4	$1289\;\mathrm{kg}/m^{3}$ [16]	13.4–19 μm

## Data Availability

Data underlying the results presented in this paper are not publicly available at this time but may be obtained from the authors upon reasonable request.
